# Centrifugally Spun Flaxseed Gum/PEO Fibers Enriched with Kale Extract for Active Food Packaging Applications

**DOI:** 10.3390/foods15152634

**Published:** 2026-07-27

**Authors:** Zulal Sila Basturk, Nalan Yazicioglu, Gulum Sumnu, Serpil Sahin

**Affiliations:** 1 Department of Food Engineering, Middle East Technical University, 06800 Ankara, Türkiye; sila.basturk@metu.edu.tr (Z.S.B.); serp@metu.edu.tr (S.S.); 2 Department of Nutrition and Dietetics, University of Health Sciences, 06018 Ankara, Türkiye; nalan.yazicioglu@sbu.edu.tr

**Keywords:** flaxseed gum, polyethylene oxide, centrifugal spinning, kale extract, lipid oxidation

## Abstract

The objective of this study was to develop active fibrous packaging materials based on flaxseed gum and polyethylene oxide containing kale extract using centrifugal spinning. In addition, their ability to retard lipid oxidation in olive oil was evaluated. Fibers containing different kale extract concentrations were produced and characterized in terms of morphology, rheological behavior, total phenolic content (TPC), antioxidant activity (AOA), encapsulation efficiency (EE), loading capacity (LC), water vapor permeability (WVP), Fourier transform infrared spectroscopy (FTIR), thermal properties, and oxidative stability. Kale extract incorporation significantly increased fiber diameter while maintaining continuous bead-free morphologies. Rheological analyses demonstrated the suitability of flaxseed gum/PEO solutions for centrifugal spinning. The incorporation of kale extract significantly enhanced TPC and AOA, whereas EE values ranged from 32.32% to 95.78%, indicating successful encapsulation of bioactive compounds. Differential scanning calorimetry (DSC), and thermogravimetric analysis (TGA) confirmed molecular interactions and adequate thermal stability of the developed fibers. Oxidative stability analyses showed that kale extract-loaded fibers effectively delayed lipid oxidation in olive oil. Among all formulations, fibers containing 5% kale extract exhibited the strongest oxidative protection, reducing p-anisidine value (PAV) and total oxidation value (TOTOX) by 25.1% and 23.86%, respectively. These findings highlight the potential of kale extract-enriched flaxseed gum/PEO fibers as sustainable active packaging materials.

## 1. Introduction

The growing demand for safer, higher-quality, and longer-lasting food products has increased interest in advanced food packaging technologies. Conventional food packaging primarily serves as a passive barrier protecting foods from environmental conditions; however, modern packaging approaches increasingly aim to actively interact with food systems in order to maintain freshness, delay spoilage, and extend shelf life. Active packaging systems incorporate functional compounds such as oxygen scavengers, antimicrobial agents, moisture absorbers, and antioxidants into packaging materials to improve food preservation performance [1,2]. Natural bioactive compounds, especially plant-derived phenolics and antioxidants, have attracted considerable attention as sustainable alternatives to synthetic additives due to increasing consumer concerns regarding chemical preservatives and environmental sustainability [1]. Lipid oxidation remains one of the major causes of quality deterioration in foods, leading to off-flavors, nutrient losses, and reduced shelf life. Thus, antioxidant-based active packaging systems have become increasingly important in food preservation applications [2].

At the same time, environmental concerns associated with petroleum-based plastics have accelerated the search for biodegradable and bio-based packaging materials. Conventional plastic packaging materials may generate persistent environmental pollution through the formation of microplastics and nanoplastics, creating significant ecological and health concerns [3]. In this context, biodegradable polymers and renewable biomaterials have emerged as promising alternatives for sustainable food packaging applications [4,5,6]. Fibrous materials produced from natural polymers have gained particular interest because of their high surface area, porosity, and loading capacity for active compounds [7,8,9]. These properties make them highly suitable for active packaging systems capable of controlling oxidation and improving food stability.

Among the various fiber production technologies, electrospinning has been extensively utilized for the fabrication of nano-fibers and active packaging materials. Nevertheless, limitations such as low production rate, high voltage requirements, and restrictions associated with polymer solution conductivity hinder its large-scale industrial application [10]. Centrifugal spinning has recently emerged as a promising alternative because it enables high productivity, does not require high voltage, and allows the processing of highly viscous polymer solutions [11]. In centrifugal spinning, the polymer jets are generated by centrifugal forces exceeding the surface tension of the spinning solution, resulting in the rapid formation of fine fibers [12]. Compared to electrospun mats, centrifugally spun fibers may provide more porous three-dimensional structures with improved thickness and loading capacity, making them especially attractive for active food packaging applications [13]. Despite these advantages, studies investigating the use of centrifugal spinning in food packaging systems remain limited. Existing studies have mainly focused on gelatin-based fibers containing natural essential oils or phenolic compounds for active packaging applications with antimicrobial and antioxidant functionalities [14,15,16].

Flaxseed gum has recently attracted increasing attention as a natural polysaccharide suitable for biodegradable packaging applications. Flaxseed gum is a water-soluble hydrocolloid extracted from the outer layer of flaxseed and mainly consists of neutral arabinoxylans and acidic rhamnogalacturonans [17]. Due to its biodegradability, film-forming ability, thermal stability, and barrier properties against oxygen transfer, flaxseed gum has been considered a promising material for edible coatings and active packaging systems [18,19]. Additionally, flaxseed gum exhibits useful functional properties such as emulsification, foaming capacity, and gel formation [20]. However, the direct spinning of flaxseed gum is challenging because of its high viscosity, water sensitivity, and poor mechanical properties [21]. To overcome these limitations, blending flaxseed gum with synthetic or semi-synthetic polymers has been suggested as an effective strategy for improving spinnability and structural stability [22].

Polyethylene oxide (PEO) is widely used in fiber production due to its excellent spinnability, hydrophilicity, biocompatibility, and low toxicity [23]. PEO can be blended with various natural polymers to improve fiber formation and rheological properties during spinning processes [23]. Previous studies demonstrated successful incorporation of PEO into fiber systems prepared from different natural biopolymers including chitosan, alginate, pea flour, and soy proteins [14,24]. Furthermore, PEO has been approved by the FDA as a safe food-contact material, supporting its potential use in food packaging systems [21]. Recent studies also showed that PEO-containing centrifugally spun active fibrous structures may effectively protect foods against oxidation and microbial deterioration [14,16,25,26].

Kale (*Brassica oleracea* var. *acephala*) is recognized as a rich source of bioactive phytochemicals including polyphenols, glucosinolates, carotenoids, lutein, and β-carotene, which contribute to its strong antioxidant activity and health-promoting properties [27,28]. The antioxidant-rich composition of kale makes it an attractive natural additive for active packaging applications intended to inhibit oxidative deterioration in foods. The high concentration of phenolic compounds and carotenoids in kale may contribute to the inhibition of lipid oxidation when incorporated into active packaging matrices [29]. Moreover, the utilization of plant-derived antioxidants in biodegradable fibrous structures may provide a dual advantage by improving food preservation while reducing dependency on synthetic packaging additives. Previous studies demonstrated that kale extract incorporated into electrospun zein fibers improved encapsulation efficiency, thermal stability, and antioxidant performance, suggesting its potential for food packaging applications [29]. However, the utilization of kale extract in centrifugally spun fibrous systems remains largely unexplored.

Although considerable research has focused on electrospun active packaging systems, studies investigating centrifugally spun flaxseed gum-based active fibers remain scarce. To the best of our knowledge, no previous study has reported the fabrication of centrifugally spun flaxseed gum/PEO fibers loaded with kale extract for active food packaging applications. Therefore, the objective of the present study was to comparatively investigate the effects of kale extract concentration on the properties of centrifugally spun active fibers developed from flaxseed gum/PEO blends. The produced fibers were further evaluated for their potential as antioxidant-active packaging materials. Olive oil was selected as a model lipid system due to its susceptibility to oxidative deterioration during storage. The produced fibers were characterized in terms of morphology, antioxidant activity, total phenolic content, encapsulation efficiency, water vapor permeability, thermal properties, and chemical interactions. In addition, their effectiveness in delaying lipid oxidation in olive oil was investigated through the determination of oxidation parameters including peroxide value, p-anisidine value, TBARS, and TOTOX values.

## 2. Materials and Methods

### 2.1. Materials

Polyethylene oxide M.W. 900 kDa was obtained from Sigma-Aldrich in St. Louis, MO, USA. The supplier of flaxseed was Mill & More Only Natural Products (Gumushane, Turkey). Sebze Meyve Dunyası Co., Inc. (Istanbul, Türkiye) supplied the kale (*Brassica oleracea*), which was sent immediately after harvest. DPPH (2,2-diphenyl-1-picrylhydrazy) and acetic acid were purchased from Sigma-Aldrich ChemieGmbh (Darmstadt, Germany). Gallic acid, sodium carbonate, ethanol, methanol, Tween 80, and Folin–Ciocalteu’s phenol reagent were all acquired from Merck (EMSURE; ACS, Reag. Ph Eur., Darmstadt, Germany).

### 2.2. Extraction of Phenolic Compounds from Kale

The preparation stage of kale for extraction was carried out with minor changes in the method of Volden et al. [30]. Initially, kale leaves were manually separated from their stems, after which the edible parts were reduced in size using a food processor (Fakir Mr. Chef Quadro, Istanbul, Türkiye). Approximately 300 g portions were subjected to blanching in 3 L of boiling water for 3 min in order to inactivate endogenous enzymes. During blanching, the processing water temperature was maintained around 95 °C. Temperature of the water during the blanching process was measured using a laboratory thermometer. Following thermal treatment, the samples were drained using a strainer and immediately cooled in ice water for 1 min, then allowed to drain for an additional minute. The blanched material was subsequently homogenized with ethanol at a ratio of 1:1 (*v*/*v*) using the same food processor. The resulting suspension was filtered three consecutive times through Whatman No. 1 filter paper to eliminate insoluble residues and to obtain a uniform liquid extract. Lastly, ethanol was removed under reduced pressure using a rotary evaporator to obtain the final kale extract.

### 2.3. Flaxseed Gum Extraction

Flaxseeds were added to 90 °C hot distilled water at a concentration of 7.7%, and the mixture was gently stirred for 10 min [20]. The gum solution after filtration using a piece of cloth was combined with three volumes of 95% ethanol for precipitation of the flaxseed gum. Centrifugation was used to gather the precipitated gum (pH 7.0), which was then dried for 5 h at 65 °C in an oven. Before being used in the studies, the dried gum was ground into fine particles using a grinder before use (Fakir Mr. Chef Quadro, Türkiye) [17].

### 2.4. Preparation of the Polymer Solutions

A polyethylene oxide (PEO) solution was obtained by dissolving PEO in acetic acid at a 3% (*w*/*v*) concentration, while the flaxseed gum solution was prepared separately by dispersing flaxseed gum in distilled water at a concentration of 0.5% (*w*/*v*). Both polymer solutions were subjected to continuous magnetic stirring at 1000 rpm and 40 °C overnight using a MaxTir 500 magnetic stirrer (Daihan Scientific, Seoul, Republic of Korea) to achieve complete solubilization. Subsequently, the two homogeneous solutions were blended in equal proportions (1:1, *v*/*v*) under the same stirring conditions for 30 min. Therefore, the final PEO/flaxseed gum-based spinning solutions contained 1.5% (*w*/*v*) PEO and 0.25% (*w*/*v*) flaxseed gum. To improve the surface properties and to promote the formation of uniform fibers, Tween 80 was added to the mixture at a final concentration of 0.8% (*w*/*v*) as a surfactant. Kale extract was incorporated into the spinning solutions at concentrations of 1%, 3%, and 5% (*w*/*v*) based on the total amount of polymers. Before centrifugal spinning, the extract was mixed into the prepared polymer solutions through magnetic stirring for 30 min. Solutions were also prepared without kale extract as a control. The fibers containing no kale extract, 1% kale extract, 3% kale extract and 5% kale extracted were denoted as PGKE-0, PGKE-1, PGKE-3 and PGKE-5, respectively.

### 2.5. Centrifugal Spinning of Fibers

The fiber fabrication procedure was adapted from the method described by Basturk et al. [31] with minor modifications. Fibers were generated at ambient temperature (25 °C) using a Nanocentrino L1.0™ system (Areka Group LLC, Istanbul, Türkiye). During the centrifugal spinning process, the rotational velocity of the spinneret was kept constant at 2400 rpm, while the solution feed rate was regulated at 15 mL h^−1^. The setup was equipped with two 23-gauge nozzles connected to the spinning chamber. For each production run, 20 mL of the prepared polymer mixture was loaded into the syringe to maintain operational consistency. A release paper collector surface with a rotation speed of 600 rpm was utilized to provide easy deposition of fibers and easy removal of the deposited fibers to avoid common problems of adhesion that are usually associated with collectors [31]. The collected fibers formed continuous self-supporting nonwoven fibrous mats, which were subsequently used for all characterization and lipid oxidation experiments.

### 2.6. Rheological Behavior of Solutions

A rheometer (Kinexus dynamic rheometer, Malvern, Worcestershire, UK) was used to evaluate the rheological properties of the PGKE-0, PGKE-1, PGKE-3, and PGKE-5 spinning solutions. The cone and plate rheometer has a 4° cone angle and a 40 mm diameter plate. Before measurements, the solution was carefully positioned, and any irregularities around the edges were corrected as needed. Shear stress data were collected at shear rates in the range of 0.1 to 100 s^−1^ at 25 °C [31].

### 2.7. Fiber Morphology Characterization

Fiber morphology was examined by field emission scanning electron microscopy (FESEM) (JEOL, Tokyo, Japan) at 2000× magnification. Fiber diameter measurements were performed using ImageJ software (Version 1.54, Bethesda, MD, USA). The average fiber diameter distribution was determined based on 50 randomly selected measurements obtained from different micrographs [31].

### 2.8. Fourier Transform Infrared Spectroscopic Analysis

The chemical structure of the kale extract-loaded fibers and their possible interactions with other components were investigated using Fourier Transform Infrared (FTIR) spectroscopy. FTIR spectra were obtained using an FTIR spectrometer (IR-Affinity1, Shimadzu, Kyoto, Japan) within the wavenumber range of 400–4000 cm^−1^ [31]. Each sample was scanned 16 times, and all analyses were carried out in duplicate.

### 2.9. Thermogravimetric Characterization

Thermal characterization of the fibers was carried out using a thermogravimetric analyzer (PerkinElmer Pyris 1, Shelton, CT, USA). Approximately 5 mg of each sample was heated from 25 °C to 600 °C at a heating rate of 10 °C/min under a nitrogen atmosphere with a gas flow rate of 30 mL/min.

### 2.10. Differential Scanning Calorimeter Analysis

Thermal properties of the fibres were measured by the DSC method (DSC Pyris 6, PerkinElmer, Waltham, MA, USA) using nitrogen as purge gas. Approximately 5 mg of each fibre was introduced in an aluminium pan, and heated in the temperature range from 25 to 300 °C with a heating rate of 5 °C/min. Reference was performed by an empty pan, and each measurement was conducted twice.

### 2.11. Evaluation of the Water Vapor Permeability of Fibers

Water vapor permeability (WVP) was determined using the gravimetric method adapted from Aydogdu et al. [32]. Cylindrical permeability cups with an internal diameter of 40 mm were utilized for the analysis. To establish 100% relative humidity inside the cups, 30 mL of distilled water was added to each container. Fiber samples were cut according to the cup dimensions, and their thicknesses were measured at six separate locations using a digital micrometer (LYK 5202, Loyka, Ankara, Türkiye). The cups were subsequently sealed with the fiber samples using rubber rings. Prior to placement in the desiccator, the initial weights of the cups were recorded. Temperature and relative humidity values were monitored for 8 h using a humidity/temperature logger (EBI20-TH1, EBRO, Ingolstadt, Germany). The average temperature and relative humidity recorded inside the desiccator during the experiments were 26.6 ± 0.7 °C and 26.1 ± 3.3% accordingly. All measurements were carried out in triplicate. The WVP values were calculated according to Equation (1). Permeability cup weights were measured after every 1 h of testing. The water vapor flux rate (G) was calculated from the slope of the linear relationship between weight loss and time in a steady state environment.

(1) $$WVP({gm}^{-1}s^{-1}{Pa}^{-1})=\frac{G\times x}{t\times A\times S\times ({R_{1}-R}_{2\;})}$$

where *G* is the water vapor flow in grams, *x* is the fiber thickness (m), *t* is time (s), *A* is the area of top surface of the cup, which was covered with fiber (m^2^), *S* is the saturated water vapor pressure in Pascal at measured temperature, *R*_1_ is relative humidity inside the cups (%), and *R*_2_ is the relative humidity inside the desiccators (%).

### 2.12. Determination of the Total Phenolic Content of Fibers

The total phenolic content (TPC) of the fibers was determined according to the method described by Aydogdu et al. [33]. Briefly, 0.4 g of fiber sample was mixed with 5 mL of ethanol:water solution (80:20, *v*/*v*). Phenolic compounds were extracted by centrifuging the mixture (Hettich Eba 20 Centrifuge; Hettich-Zentrifugen Holding GmbH & Co., Tuttlingen, Germany) at 5000 rpm for 10 min. Subsequently, 0.5 mL of the diluted extract was mixed with 2.5 mL of 0.2 N Folin–Ciocalteu reagent and vortexed (ZX3; VELP Scientifica, Usmate, MB, Italy) for 1 min. The resulting solution was filtered using a syringe filter (ISOLAB/PTFE 45/25), followed by the addition of 2 mL sodium carbonate solution (7.5%, *w*/*v*). The mixture was then kept in the dark for 1 h before measuring its absorbance at 760 nm using a UV/VIS spectrophotometer (Shimadzu UV 2450, Columbia, MD, USA). A standard calibration curve was generated using gallic acid solutions prepared in ethanol:water (80:20, *v*/*v*) at different concentrations. TPC values were calculated according to Equation (2) and reported as milligrams of gallic acid equivalent (GAE).

(2) $$TPC(\frac{\mathrm{mg}\;\mathrm{GAE}}{g\;\mathrm{film}})=\frac{C\times V\times D}{W_{s}}$$

where *C* represents the solution concentration determined from the calibration curve (mg/L), *W_s_* is the fiber weight (*g*), *D* is dilution ratio, and *V* is fiber solving solution volume (*L*).

### 2.13. DPPH Radical Scavenging Activity of Fibers

Antioxidant activity was determined using the 2,2-diphenyl-1-picrylhydrazyl (DPPH) radical scavenging assay according to the method reported by Aydogdu et al. [33]. For the extraction procedure, 0.4 g of fiber sample was dispersed in 10 mL of ethanol:water mixture (80:20, *v*/*v*). The prepared mixture was centrifuged at 5000 rpm for 10 min using a Hettich-Zentrifugen centrifuge (Tuttlingen, Germany). Following centrifugation, the collected supernatant was combined with a 25 ppm DPPH solution and mixed thoroughly using a vortex mixer. The reaction mixture was then stored in the dark for 30 min prior to absorbance measurement at 517 nm using a UV/VIS spectrophotometer (Shimadzu UV2450, Columbia, MD, USA). The antioxidant activity values were calculated based on Equation (3).

(3) $$\mathrm{Antioxidant}\;\mathrm{Activity}\left(\%\right)=\frac{A_{control}-A_{sample\;}}{A_{control}}\times 100$$

where the absorbance of the fiber-containing DPPH solution is *A_sample_,* and the absorbance of the fiber-free DPPH solution is *A_control_*.

### 2.14. Determination of Encapsulation Efficiency and Loading Capacity

Encapsulation efficiency (EE) and loading capacity (LC) were determined using a modified method based on Atencio et al. [34]. The theoretical TPC of each formulation was calculated based on the experimentally determined TPC of the kale extract and the amount of extract added to the polymer solution. The measured TPC of the kale extract-containing fibers was determined by extracting the phenolic compounds from the fibers and measuring their TPC using the Folin–Ciocalteu assay. The measured TPC values were corrected by considering the solid content of the flaxseed gum/PEO spinning solution. Finally, Equations (4) and (5) were used to calculate EE and LC, respectively.

(4) $$EE\left(\%\right)=\frac{Measured\;TPC\;of\;kale\;extract\;containing\;fibers}{\mathrm{Theoretical}\;\mathrm{TPC}\;\mathrm{contributed}\;\mathrm{by}\;\mathrm{the}\;\mathrm{incorporated}\;\mathrm{kale}\;\mathrm{extract}}\times 100$$

(5) $$LC\left(\%\right)=\frac{Measured\;TPC\;of\;kale\;extract\;containing\;fibers}{Weight\;of\;fibers\;used\;for\;the\;TPC\;analysis}\times 100$$

### 2.15. Assessment of Lipid Oxidation

Petri dishes containing 4 g of refined olive oil and 0.3 g of fiber samples were prepared for lipid oxidation analysis. Four different fiber formulations were evaluated, including a control fiber without kale extract and centrifugally spun fibers containing 1%, 3%, and 5% kale extract. The prepared Petri dishes were stored in a dark incubator (Nuve NO55, Ankara, Türkiye) at 40 °C for 14 days. Peroxide value (PV), p-anisidine value (PAV), and thiobarbituric acid reactive substances (TBARS) analyses were conducted on days 1, 7, and 14 of storage with two replicates for each sample.

Peroxide value (PV) analysis was performed according to the method described by Chong et al. [35]. Briefly, 0.1 g of oil sample was mixed with 0.6 mL of an acetic acid:chloroform solution (2:3, *v*/*v*) to facilitate dissolution of the oil phase. Subsequently, 0.01 mL of saturated potassium iodide solution was added, and the mixture was shaken for 1 min. Afterwards, 0.6 mL of distilled water and 0.02 mL of starch solution (1 g/100 mL water) were incorporated into the mixture. The resulting solution was titrated with 0.005 N sodium thiosulfate until a transparent endpoint was reached. The peroxide value was then calculated using the following equation.

(6) $$PV\;(mEq\frac{peroxide}{\mathrm{kg}\;\mathrm{of}\;\mathrm{oil}})=\frac{\left(S-B\right)\times N\times 100}{W}$$

where *S* is the titrant volume of the sample (mL), *B* is the titrant volume for the blank (mL), *N* is the normality of the Na_2_S_2_O_3_ solution, and *W* is the sample weight (g).

The p-anisidine value (PAV) was determined following the method reported by Chong et al. [35]. For the analysis, 0.1 g (m) of oil sample was dissolved in 5 mL of isooctane, and the absorbance of the solution was measured at 350 nm using a UV–VIS spectrophotometer (T80+, UV/Vis spectrometer, PG Instrument Ltd., Lutterworth, UK), recorded as *A*_1_. Subsequently, 1 mL of 0.25% p-anisidine solution was added to the sample, which was then incubated in the dark for 10 min. After incubation, the absorbance at 350 nm was measured again and recorded as *A*_2_. A control sample containing isooctane instead of the oil sample was used as the blank (*A_k_*). The PAVs were calculated using the following equation.

(7) $$PAV=\frac{25\times [(1.2\times \left(A_{2}-A_{k\;}\right)-A_{1}]}{m}$$

Thiobarbituric acid reactive substances (TBARS) analysis was carried out by weighing 20–80 mg of olive oil into sample tubes and adjusting the total volume to 5 mL with 1-butanol. The 2-TBA reagent solution was prepared by dissolving 50 mg of 2-thiobarbituric acid in 25 mL of 1-butanol [36]. Subsequently, equal volumes (2 mL) of the sample solution and TBA reagent were mixed thoroughly. The prepared mixtures were heated in a water bath at 95 °C for 2 h. Following cooling to room temperature, absorbance values were measured at 532 nm using a UV/VIS spectrophotometer (*A_s_*). TBARS values were calculated using the following equation and expressed as mg malondialdehyde (MDA) per gram of oil. The term *A_B_* corresponds to the absorbance value of the blank sample.

(8) $$TBARS=\frac{50\times (A_{s}-A_{B})}{m}$$

where m is the weight of olive oil. The total oxidation (TOTOX) values of the oil samples were calculated using the relevant equation, based on the PAV and PV data acquired, as described by Chen et al. [37].

(9) TOTOX=2PV+PAV

### 2.16. Statistical Evaluation

Statistical analyses were performed using MINITAB software (Version 19, State College, PA, USA). The experimental data were evaluated by analysis of variance (ANOVA), and Tukey’s multiple comparison test was employed to identify significant differences among samples at a significance level of *p* < 0.05. All experiments were conducted with a minimum of three replicates.

## 3. Results

### 3.1. Rheological Properties of Solutions

The rheological characteristics of the polymer solution serve as a primary determinant of the intermolecular interactions among polymer chains, which are crucial for governing both the spinnability of the solution and the resulting fiber morphology. When polymer solutions exhibit very high viscosity, the enhanced intermolecular bonding among polymer chains restricts their movement, making jet formation unstable reducing the efficiency of fiber production significantly [23].

**Figure 1:**
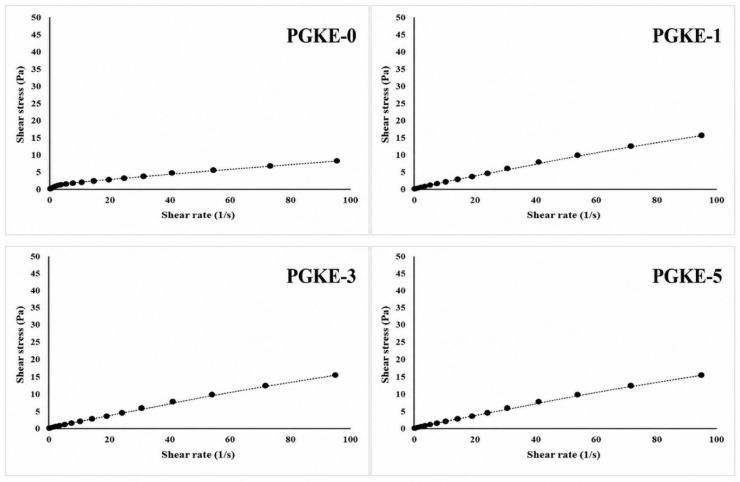
Shear stress versus shear rate curves of polymer solutions containing different concentrations of kale extract.

In the present study, all of the polymer solutions exhibited Newtonian flow behavior within the investigated shear rate range (0.1–100 s^−1^), as indicated by linear shear stress–shear rate relationships and high coefficients of determination (R^2^ = 0.99) (Figure 1). Similar Newtonian behavior has been reported for rice bran protein/flaxseed gum solutions [38]. Also, according to another study, olive leaf polyphenols were incorporated into a sweet almond gum and gelatin blend using electrospinning, where the gum addition led to Newtonian-type flow behavior [38].

Flaxseed gum is a polysaccharide capable of forming intermolecular interactions through hydrogen bonding [17]. Similar behavior has previously been reported for dilute polysaccharide-based spinning systems, where limited intermolecular entanglement resulted in relatively stable flow properties under shear conditions.

However, the relatively low flaxseed gum concentration used in the present study may have limited the formation of extensive chain entanglements within the polymer system. As a result, the viscosity remained relatively stable throughout the investigated shear rate range, leading to flow behavior close to Newtonian characteristics in flaxseed gum containing solutions [17]. Although high-viscosity polymer solutions are often associated with non-Newtonian behavior [23], Newtonian characteristics may still be observed depending on polymer concentration, solvent characteristics, and processing conditions [23]. Moreover, near-Newtonian flow behavior may contribute to stable jet formation during centrifugal spinning under high rotational forces [23].

The viscosity values of solutions are presented in Table 1. No statistically significant differences were observed among samples with different kale extract concentrations, suggesting that the relatively narrow concentration range of kale extract (1–5%) was insufficient to change the rheological properties of the polymer system. Increasing the extract concentrations did not result in significant differences among the active substance containing samples, whereas the control sample (0% PE) differed significantly from all extract containing formulations [24].

The incorporation of kale extract significantly increased viscosity compared with PGKE-0. The viscosity of the kale extract-containing formulations in the present study ranged between 0.132 and 0.209 Pa·s, which was lower than the viscosity values previously reported for centrifugally spun gelatin-based fibers containing the same kale extract concentrations (0.91–1.67 Pa·s) [31]. Gelatin solutions (25% *w*/*v*) generally exhibit higher viscosity due to strong intermolecular interactions, extensive chain entanglement, and protein network formation in solution. In contrast, the flaxseed gum/PEO formulations used in the study contained lower effective polymer concentrations and weaker intermolecular entanglement density, resulting in lower overall viscosity values despite successful fiber formation.

### 3.2. Morphology of Fibers

**Figure 2:**
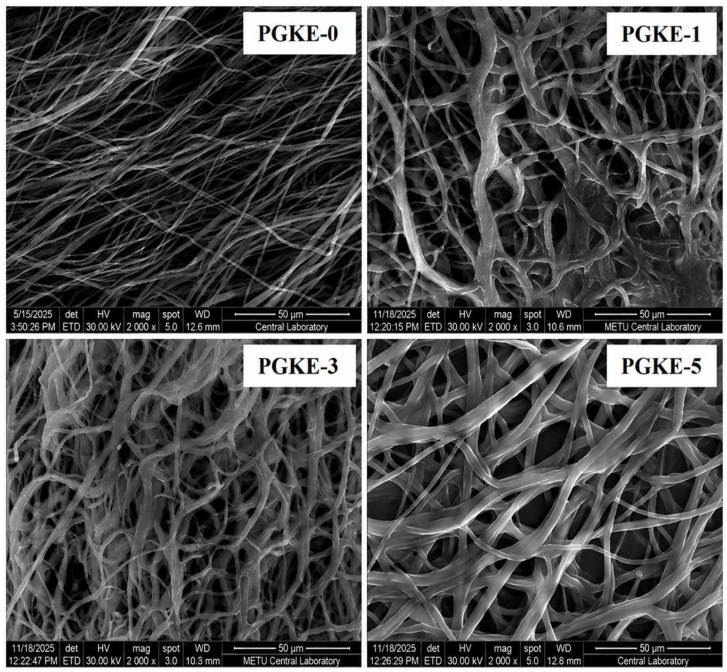
FESEM images of flaxseed gum/PEO-based fibers containing different concentrations of kale extract.

**Figure 3:**
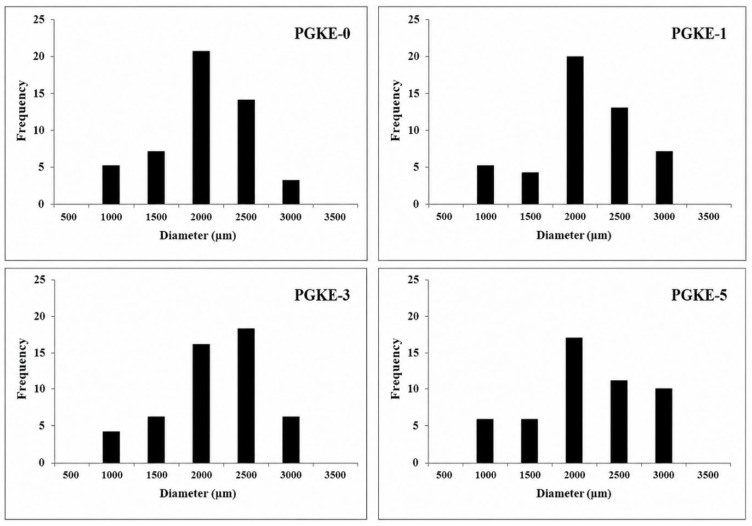
Diameter distribution of flaxseed gum/PEO-based fibers containing different concentrations of kale extract.

Centrifugally fabricated fibers exhibited randomly distributed continuous fibers with bead-free morphologies, indicating successful fiber formation during centrifugal spinning (Figure 2). The corresponding fiber diameter distributions for each formulation are presented in Figure 3. Surface tension exerts a fundamental influence on the spinnability of polymeric solutions, while viscosity also plays a key role in determining fiber formation quality. During spinning, the solution must experience sufficient force at the needle tip to surpass surface tension, allowing the jet to form and extend toward the collector. Sustaining this force throughout the trajectory ensures the creation of continuous and uniform fibers [39]. Fiber morphology was influenced by kale extract incorporation. Average fiber diameters increased significantly with kale extract incorporation. All formulations were successfully collected as continuous nonwoven fibrous mats. While no significant difference was observed between PGKE-1 and PGKE-3, PGKE-5 exhibited the highest diameter among all formulations (Table 1).

The increase in fiber diameter may be explained by the interaction of kale extract molecules with the PEO polymer chains, which can influence solution properties and jet stretching behavior during centrifugal spinning [24]. The addition of kale extract resulted in significantly higher fiber diameters relative to the PGKE-0 fibers. Although PGKE-1 and PGKE-3 were statistically similar, PGKE-5 exhibited a significantly greater fiber diameter, indicating that higher extract loading contributed to fiber thickening. A similar result was obtained in the study of fabrication of polylactide fibers via electrospinning, containing *Portulaca oleracea* plant extract as an active agent [40]. The inclusion of the extract led to larger mean fiber diameters relative to the control formulation [40]. Bioactive compounds in kale extract may interact with PEO chains through hydrogen bonding and other intermolecular interactions, reducing jet stretching during spinning and resulting in thicker fibers. These findings indicated that the highest kale extract concentration tested (5%) promoted greater fiber diameter development.

### 3.3. Water Vapor Permeability

In food packaging, the water vapor permeability (WVP) of fibers serves as a key parameter in determining their effectiveness as moisture barriers. Low WVP values are advantageous because they limit the loss of water from food products into the surrounding atmosphere [33]. WVP through the film is mainly attributed to its hydrophilic surface, and the extent of this transfer is strongly shaped by the equilibrium between hydrophilic and hydrophobic interactions [41]. Table 1 presents the WVP values of the fibers, which range from 4.78  ±  0.191 to 6.66  ±  0.375  ×  10^−12^ g·m^−1^·s^−1^·Pa^−1^. PEO/flaxseed gum fibers without kale extract (PGKE-0) exhibited the lowest WVP values, indicating a more compact structure and better resistance to water vapor transfer. In contrast, all samples containing kale extract showed higher WVP values. This increase can be attributed to the hydrophilic nature of the bioactive compounds present in the kale extract, which might have enhanced the affinity of the fiber matrix for water molecules and facilitated water vapor diffusion [42]. The absence of significant differences among PGKE-1, PGKE-3, and PGKE-5 suggested that once kale extract was incorporated into the polymer matrix, further increases in extract concentration had a limited additional effect on water vapor permeability. The increase in fiber diameter after kale extract incorporation might also have contributed to higher WVP values by altering the packing density and porosity of the fibrous structure. Similarly, in the study where the grape seed extract was incorporated in electrospun rye flour/whey protein/PEO nanofibers, the water vapor permeability of the fiber films increased as extract was added [42]. The increase in WVP may be associated with interactions between the components of kale extract and the flaxseed gum/PEO matrix. However, this did not result in improved water vapor barrier properties, likely due to the hydrophilic nature of the phenolic compounds, which may facilitate moisture transport [43]. In comparison with previously reported centrifugally spun gelatin-based kale extract-loaded fibers, the WVP values of the present PEO/flaxseed gum-based fibers were slightly higher [31]. This difference might be attributed to the more hydrophilic nature of PEO and flaxseed gum, which could increase the water affinity within the fibrous matrix and facilitate water vapor transfer. In both studies, the incorporation of kale extract increased WVP compared with the extract-free control, indicating that kale-derived bioactive compounds might enhance moisture transport due to their hydrophilic phenolic constituents.

### 3.4. Total Phenolic Content (TPC) & Antioxidant Activity (AOA)

The total phenolic content in kale extract containing fibers ranges from 201.67 to 1167 mg per 100 g [44,45]. A major subclass, hydroxycinnamic acids, which are cinnamic acid derivatives with 3–6 carbon structures, act as powerful antioxidants and play a crucial role in maintaining flavor, color stability, and nutritional quality in foods reported that kale contains around 204 mg hydroxycinnamic acids per 100 g fresh weight [27]. Previous studies have reported that kale is also a rich source of glucosinolates, secondary metabolites typical of the Cruciferae family, with levels measured at 41 µmol/g on a dry weight basis. The incorporation of kale extract led to concentration-dependent increase in TPC values in the PEO/flaxseed gum-based fibers. Among the kale extract-containing samples, PGKE-1 exhibited the lowest TPC, while PGKE-3 and PGKE-5 showed progressively higher values. This trend confirmed that increasing kale extract concentration effectively enhanced the phenolic content of the fibers. Although PGKE-0 did not contain kale extract, low but detectable TPC and AOA values were observed. This may be attributed to naturally occurring phenolic compounds present in flaxseed gum, including phenolic acids and lignan derivatives that can remain associated with the polysaccharide matrix after extraction [17]. In addition, the higher TPC values observed in the present study compared to the gelatin-based fibers [31] might indicate that the flaxseed gum/PEO matrix provided a more favorable environment for retaining and protecting phenolic compounds during centrifugal spinning. The polysaccharide-rich structure of flaxseed gum might have promoted hydrogen bonding and reduced the degradation or loss of antioxidant compounds during fiber formation. Likewise, when Pinhão (*Araucaria angustifolia*) coat extract was incorporated into starch fibers by electrospinning at concentrations of 0–1.5% (*w*/*v*), total phenolic content increased progressively with increasing extract concentration [46]. Similarly, a study reported that increasing the incorporation level of pomegranate flower extract into sage seed gum/gelatin multilayer films resulted in an increase in total phenolic content [43].

Rich in health-promoting phytochemicals such as vitamin C, carotenoids, flavonoids, and diverse phenolics, kale is recognized for its pronounced antioxidant activity. When compared with other members of the *Brassica oleracea* group, it shows superior antioxidant potential [27]. As presented in Table 2, the presence of antioxidant activity in PGKE-0 further supports the contribution of flaxseed gum derived phenolic compounds [17]. However, the substantial increase observed with increasing kale extract concentration indicated that kale extract was the primary source of antioxidant capacity in the developed fibers. As the kale extract concentration increased from 1% to 5%, AOA increased significantly, with PGKE-5 exhibiting the highest antioxidant activity. This trend closely followed the increase observed in total phenolic content, indicating that phenolic compounds from kale extract were likely the primary contributors to antioxidant activity.

### 3.5. Encapsulation Efficiency (EE) & Loading Capacity (LC) Analysis

The encapsulation efficiency (EE) of fibers produced through centrifugal spinning is influenced by several parameters, including the setup of the spinning process, the polymer type and its molecular weight, as well as the distribution of fiber diameters [47]. Furthermore, EE is determined by the physicochemical interactions between the encapsulated compound and the polymer matrix, which are influenced by the chemical properties of both materials. High levels of encapsulation efficiency are important for ensuring the stability of bioactive substances, as they help to protect bioactive compounds from direct environmental impact [48]. The encapsulation efficiencies of fibers containing 1%, 3%, and 5% (*w*/*w*) PGKE were determined as 95.78 ± 0.55%, 46.55 ± 0.52%, and 32.32 ± 0.83%, respectively. The results demonstrated that increasing the concentration of kale extract decreased EE while increasing LC values. A reduction in EE with increasing concentrations of bioactives is a commonly observed phenomenon, often attributed to insufficient incorporation of bioactive compounds at higher loadings or to inherent limitations in the capacity of the matrix to accommodate them [49]. Relative to the gelatin-based fibers from the previous work [31], the PEO/flaxseed gum system exhibited improved kale extract encapsulation performance.

In food applications, the effectiveness of encapsulating bioactive molecules largely depends on the loading capacity, which guarantees their stability and availability at levels sufficient to exert the expected biological activity [34]. This parameter is influenced by the physicochemical features of the core and wall materials and the interactions between them [34]. As can be seen in Table 2, LC values increased inversely with the EE value as the kale extract concentration increased. Similarly, the LC value of catechin-loaded electrospun nanofibers from Azivash gum/PVA increased with the increased concentration of catechin [50]. Compared with the gelatin-based fibers in the earlier study [31], the loading capacity values were marginally higher, which was consistent with the slightly elevated TPC values observed in the current fiber system.

### 3.6. FTIR

**Figure 4:**
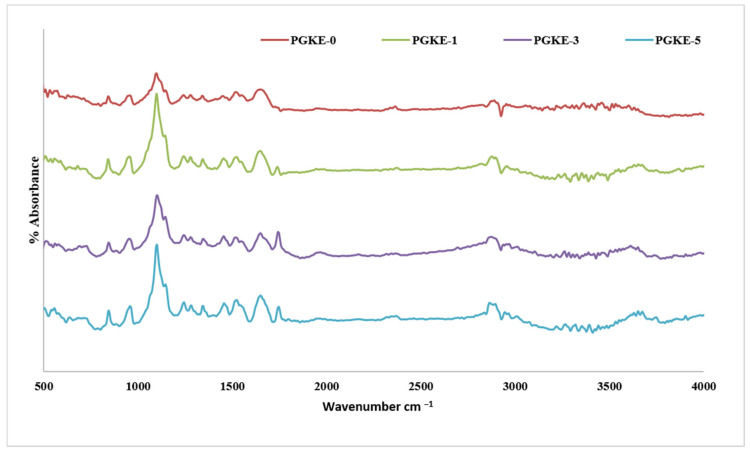
FTIR spectra of centrifugally spun fibers.

To investigate the chemical functionalities and intermolecular bonding within the mixtures, FT-IR spectroscopy was utilized. The obtained spectra for flaxseed gum/PEO fibers containing different concentrations of kale extract are shown in Figure 4. The band detected around 842 cm^−1^ reflects C–O–C and C–C stretching vibrations of the flaxseed gum/PEO matrix. Peaks observed near 960, 1100 and 2890, were mainly associated with ether and CH_2_ stretching vibrations of the PEO containing polymer matrix, while the broad absorption around 3461 cm^−1^ was attributed to O–H stretching related to absorbed water, flaxseed gum hydroxyl groups, and phenolic compounds from kale extract [51]. Slight changes in peak intensity were observed in kale extract-containing fibers compared with the control formulations. Prominent peaks around 2800–2900 cm^−1^ are assigned to CH_2_ symmetric and asymmetric stretching vibrations, which are characteristic of aliphatic chains present in kale-derived compounds such as cutin and wax constituents [49]. No substantial changes were observed in the intensity of peaks within this region after kale extract incorporation, suggesting that the main aliphatic structure of the fibers was preserved [28]. This indicates that as the proportion of kale extract rises, the encapsulation efficiency tends to decrease. A distinct band observed around 1739 cm^−1^ can be attributed to C=O stretching vibrations, characteristic of carbonyl-containing compounds [52]. A small peak appearing around 1149 cm^−1^ can be attributed to O–C–O asymmetric stretching vibrations, which indicate the occurrence of xyloglucan structures related to flaxseed gum [53]. The absorption band located at approximately 1620 cm^−1^ is attributed to the asymmetric C=O stretching vibration of carboxyl groups, which may be associated with absorbed or bound water molecules within the flaxseed gum matrix. Such carboxyl functionalities interact with ions and have a marked effect on the gum’s gelling potential and rheological behavior [54]. The spectral feature located at approximately 1415 cm^−1^ arises from C–O stretching vibrations, which are characteristic of uronic acid functionalities [55]. The interval between 800 and 1200 cm^−1^ is recognized as the fingerprint region specific to carbohydrates, offering an effective means of detecting structural distinctions among different types of gums [56]. Furthermore, a characteristic peak around 835 cm^−1^ was associated with mannose residues and α-D-galactose structures within the polysaccharide matrix [57]. A characteristic peak around 835 cm^−1^ corresponds to α-D-galactose, indicating its presence within the sample structure. Crystallinity in gum-based materials is commonly identified within the 700–500 cm^−1^ spectral interval [58]. Therefore, the peak appearing around 687 cm^−1^ in the flaxseed-gum-added fibers can be attributed to the crystalline domains present in the biopolymer [52]. Results suggest that kale extract was successfully incorporated into the fibrous matrix without substantially altering the main chemical structure of the polymers. The observed spectral changes may indicate intermolecular interactions such as hydrogen bonding between kale-derived phenolic compounds and the polymer matrix.

### 3.7. Thermogravimetric Analysis and Differential Scanning Calorimetry

**Figure 5:**
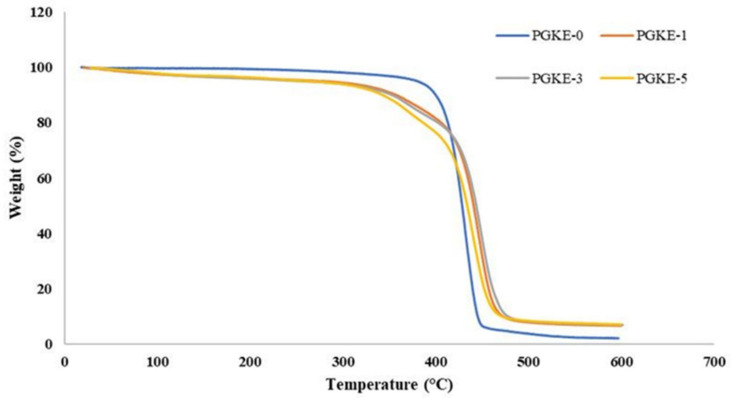
Thermogravimetric curves of flaxseed gum-based samples.

Thermogravimetric analysis was used to clarify the heat stability of the fibers. TGA and DSC analyses were conducted primarily to assess the thermal behavior of the flaxseed gum/PEO-based composite fibers. The weight loss versus temperature curve for the heated fibers was shown in Figure 5, while the corresponding thermal degradation stages and DSC parameters are summarized in Table 3. With the exception of the control sample (PGKE-0), which was lacking kale extract, all of the composite fiber membranes showed comparatively similar thermal degradation characteristics. According to the figure, weight loss occurred between 50 and 100 °C and between 330 and 480 °C. The volatilization of bound moisture and acetic acid molecules was responsible for the first stage weight loss, which lasted from 50 °C to 100 °C [51]. In this range, a weight loss of 1.51%, 1.56% and 1.48% was observed in PGKE-1, PGKE-3 and PGKE-5 fibers, respectively. In the control fiber (PGKE-0), there was no volume loss in this range, but as a result, no significant effect was observed on thermal degradation in the first stage of the addition of the kale extract. In the second heat degradation range between 330 °C and 480 °C, the weight loss for PGKE-0, PGKE-1, PGKE-3 and PGKE-5 was 95.22%, 90.38%, 89.62% and 89.89%, respectively. The thermal degradation of PEO is responsible for the second stage’s extension from 330 to 480 °C [59]. Weight reduction from PGKE-0, PGKE-1, PGKE-3, and PGKE-5 was 97.79%, 93.24%, 92.94%, and 92.70%, respectively. At the end of the analysis, fibers from kale-extracted samples still contained around 7% of the weight. There was just about 2% of fiber left in the control fiber. The reason for this might be due to the fact that PGKE-0 was less dense because kale extract was not added. Furthermore, out of all the fibers created, the PGKE-5 performed the best in terms of thermal stability. Similar results were obtained in the investigation of solution blowing spun PEO/Sesbania gum fiber when combined with ε-poly(lysine) [59]. TGA investigation showed that the thermal stability of the kale extract-added PEO/flaxseed gum-based fibers improved considerably which was important in terms of its application in food-active packaging.

Table 3 displays the DSC results of fibers with and without kale extract as enthalpies (ΔH_m_) and melting temperatures (T_m_). The thermal characteristics of solid materials, including melting temperatures and enthalpy values, are quantified and qualitatively reported by DSC [60]. Based on the amount of heat needed to melt the crystalline material, DSC offers a quick way to calculate the degree of crystallinity. In other words, the sample’s crystallinity determines the enthalpy of a melting process [61]. The enthalpies and melting temperatures ranged from 18.52 to 122.58 J/g and 52.84 to 66.83 °C, respectively. There was an observation of lower values for the melting enthalpies and melting temperature at higher kale extract contents, signifying the presence of lower crystallinity in the polymer network. This effect might be due to the interaction between the intermolecular bonding, the triple helix structure, and the presence of the phenolic compounds found in the kale extract, which reduces the stability of the crystalline structures [61].

Compared with previously reported gelatin-based kale extract-loaded fibers [31], the PEO/flaxseed gum-based fibers exhibited lower residual mass at elevated temperatures (approximately 7%) than the gelatin-based fibers (approximately 24%) but showed higher thermal degradation onset temperatures. This difference may be attributed to the distinct thermal decomposition behaviors of protein-based and polysaccharide/PEO-based system. In the gelatin-based system, kale extract may be acted as a filler within the protein matrix, whereas in the present PEO/flaxseed gum system, kale extract may show an effect on the polymer organization. The decreases in melting temperature (T_m_) and melting enthalpy (ΔH_m_) after kale extract incorporation indicate that the polymer structure became less ordered and more flexible. This may be related to interactions between kale-derived phenolic compounds and the polymer chains, which likely interfered with intermolecular packing and reduced the formation of crystalline regions within the fibers.

### 3.8. Lipid Oxidation

It is well known that lipid oxidation, which occurs when lipids with any number of C single bonds or C double bonds in foods containing lipids, not only leads to the production of hazardous and unhealthy secondary reaction products, but also to a decline in organoleptic quality and shelf life, all of which affect food quality and increase safety. It is also directly linked to an increase in food waste and financial losses [62]. By measuring the amount of secondary volatile chemicals including alcohols, ketones, and aldehydes as well as primary oxidation products like hydroperoxides, the oxidative stability of the generated fibers was assessed [62]. Oxidation product concentrations have demonstrated clear time-dependent rises with differing rise degrees in all samples based on the extension of storage duration. An increase in lipid oxidation over seven days occurred due to the breakdown of O–O bonds in lipid hydroperoxides, leading to the generation of highly reactive intermediate radicals, namely alkoxyl and peroxyl [63].

**Figure 6:**
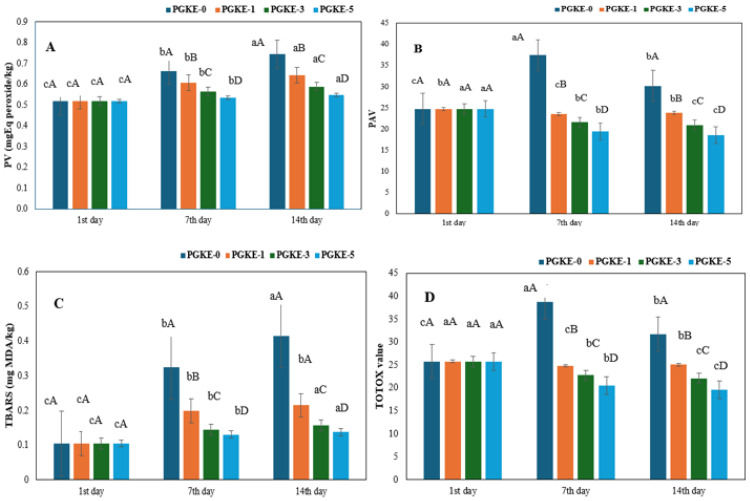
Oxidation parameters of olive oil samples containing flaxseed gum-based fibers with different kale extract concentrations (PGKE-1, PGKE-3, and PGKE-5) and control fibers (PGKE-0): (**A**) peroxide value (PV), (**B**) p-anisidine value (PAV), (**C**) TBARS, and (**D**) TOTOX values measured on days 1, 7, and 14. Different lowercase letters indicate significant differences among storage days [NY9.1] within the same formulation, whereas different uppercase letters indicate significant differences among formulations on the same storage day (*p* < 0.05).

PV serves as an essential index for monitoring the early oxidation phase of lipids, where hydroperoxides are initially produced. Despite their relative stability at this stage, these primary oxidation species can later decompose into secondary molecules capable of impairing sensory attributes and potentially exerting adverse health effects, contributing to rancidity development [64]. The Codex Standard specifies a maximum permissible peroxide value of 10 meq O_2_/kg for refined edible oils to remain within acceptable quality limits [65]. The olive oil samples containing kale extract fibers that were subjected to 14 days of heat treatment exhibited PV levels exceeding this established limit. As shown in Figure 6A, PV of the oil samples at the beginning of the experiment was 0.523 ± 0.007 meq O_2_/kg. Throughout the two-week accelerated storage study, PVs increased steadily. All the formulations with kale extract (PGKE-1, PGKE-3, and PGKE-5) had lesser values of peroxide compared to the control formulation (PGKE-0) during the entire storage period, which implies that the kale extract inhibits the formation of primary oxidation compounds. The more the amount of kale extract added, the more effective it is. At the end of the testing period, oils packaged with PGKE-5 fibers reached a PV of 0.559 ± 0.0172 meq O_2_/kg, whereas the control samples rose to 0.754 ± 0.0142 meq O_2_/kg. These results indicated that PGKE-5 fibers substantially limited peroxide accumulation. The elevated kale extract content in the fibers also enhanced both TPC and AOA, which likely contributed to suppressing the rise in PV and keeping it below levels associated with oxidative deterioration. Compared with the control formulation (PGKE-0), the PGKE-5 formulation exhibited a 23.33% reduction in peroxide value, indicating its higher effectiveness in preventing primary lipid oxidation. Overall, the data demonstrated that kale extract provided strong radical-scavenging capacity and protection against lipid oxidation.

p-Anisidine value (pAV) is used to evaluate secondary lipid oxidation products, particularly aldehyde compounds formed during the decomposition of hydroperoxides in oils and fats [66]. Exposure of the oils to heat caused the breakdown of hydroperoxide intermediates into multiple secondary molecules, such as esters, aldehydes, alkenals, and dienals. The formation of these oxidation by-products adversely affects the sensory profile, nutritional composition, and general acceptability of food products [66]. As an indicator of secondary oxidative degradation, p-AV reflects the concentration of reactive compounds such as α- and β-alkenals that interact with p-anisidine. Consequently, a thorough assessment of lipid oxidation involves monitoring both early-stage and advanced oxidation products formed under high-temperature conditions [67]. At the beginning of the analysis, the p-anisidine value (pAV) of the oil samples was determined to be 25.086 ± 0.46. Across all formulations, the p-anisidine values (pAV) showed a continuous upward trend throughout the 14-day storage experiment (Figure 6B). When compared with the control sample, fibers containing kale extract (PGKE-1, PGKE-3, and PGKE-5) demonstrated a reduction in lipid oxidation intensity. On day 14, the p-anisidine level of the oil containing control fibers reached nearly the same magnitude as that measured after the initial week, suggesting a consistent progression of secondary oxidation. In contrast, among the kale extract incorporated fibers, the PGKE-5 treatment exhibited the least rise in PAV. Following 14 days of storage, decreases in p-anisidine value were recorded as 3.95% for PGKE-1, 15.44% for PGKE-3, and 25.1% for PGKE-5 as compared to control. The presence of kale extract appears to suppress or postpone the generation of α-unsaturated aldehydes, likely due to the activity of its phenolic content that influence oxidative reactions [68]. Comparable findings were reported for tannic acid, as guar gum-based fibers likewise exhibited antioxidant activity that contributed to slowing oxidative reactions in flaxseed oil [21].

Malondialdehyde (MDA) concentration, determined through TBARS analysis, serves as an indicator of lipid oxidative degradation. As storage progresses, the unsaturated lipids within the oil undergo oxidative reactions and decomposition, yielding MDA as a secondary product [69]. Higher TBARS measurements correspond to a more advanced stage of lipid peroxidation [70]. TBARS levels above 0.5 mg/kg are typically linked to observable off-flavors [70]. By the seventh day of storage, the TBARS measurements for the olive oil samples packaged with the various fibers did not differ significantly and remained in line with the values obtained on day one. In contrast, the control samples without kale extract showed a pronounced rise in TBARS, as illustrated in Figure 6C. Over the two-week period, the fiber sample without extract exhibited a greater accumulation of secondary oxidation products than the kale-extract-enriched fibers. When compared to PGKE-0, the fiber formulations supplemented with kale extracts (PGKE-1, PGKE-3, and PGKE-5) showed a decrease in the formation of TBARS after 14 days of storage, suggesting that the lowest level of kale extracts was also protective. Among the formulations, the fibers containing 5% kale extract displayed the smallest increase in TBARS relative to the 3% and 1% extract fibers. On the 14th day, PGKE-5 reduced TBARS values by approximately 66.93% compared with the control sample, indicating the strong inhibitory effect of kale extract against secondary lipid oxidation products. A comparable trend in TBARS reduction was reported in research where zein-based fibers enriched with saffron (*Crocus sativus* L.) extract were applied as coverings for sea bass fillets [71].

TOTOX value, calculated from peroxide value (PV) and p-anisidine value (pAV), provides a comprehensive assessment of both primary and secondary lipid oxidation products in oils. Variations in the Totox value were directly influenced by storage conditions and time [72]. As shown in Figure 6D, TOTOX values increased during the 14-day accelerated storage period, indicating oxidative deterioration of olive oil samples. However, the increase was substantially higher in the control formulation (PGKE-0) than in the kale extract-loaded fiber formulations. This difference likely stems from the protective role of antioxidants, which intercept free radicals and hinder the initiation of lipid autoxidation. Incorporating kale extract into gelatin-based fibers lowered total olive oil oxidation by approximately 11.03% [31]. In contrast, the PEO/flaxseed gum kale extract fibers achieved a 23.86% decrease, which may be attributed to variations in polymer composition or experimental parameters. The disparity in oxidation reduction between the earlier study and the present work is most likely attributable to differences in the polymers utilized. Although both investigations applied the same centrifugal spinning technique and incorporated the same active compound, the distinct polymer matrices appear to have influenced the final outcomes. Overall, all kale extract-loaded fibers (PGKE-1, PGKE-3, and PGKE-5) lowered lipid oxidation relative to the control, with PGKE-5 producing the most pronounced reduction. This effect corresponded to the higher antioxidant activity observed in the PGKE-5 formulation.

## 4. Conclusions

Within the scope of this study, active flaxseed gum/PEO-based fibers containing different concentrations of kale extract were successfully fabricated through centrifugal spinning for potential active food packaging applications. The incorporation of flaxseed gum and kale extract influenced the physicochemical, structural, and functional properties of the fibers while maintaining continuous and bead-free morphologies suitable for fiber formation. The incorporation of kale extract significantly increased the average fiber diameter while maintaining continuous and bead-free morphologies suitable for packaging applications. Rheological analysis demonstrated that flaxseed gum/PEO spinning solutions possessed appropriate flow behavior for stable centrifugal spinning and fiber formation. The incorporation of kale extract markedly enhanced the antioxidant activity and total phenolic content of the fibers owing to the bioactive phenolic compounds naturally present in kale extract. Encapsulation efficiency analysis confirmed the successful incorporation of phenolic compounds into the fibrous structures. FTIR and thermal analyses further demonstrated the compatibility and stability of the developed fiber systems. The developed fibers exhibited promising oxidative protection performance during olive oil storage. All kale extract-containing fiber formulations (PGKE-1, PGKE-3, and PGKE-5) improved the oxidative stability of olive oil compared with the control formulation (PGKE-0), demonstrating the antioxidant effectiveness of kale extract irrespective of concentration. Among all formulations, PGKE-5 demonstrated the strongest protective effect by exhibiting the lowest peroxide value, p-anisidine value, TBARS, and TOTOX values throughout accelerated storage. These findings indicated that increasing the kale extract concentration enhanced the oxidation inhibitory performance of the fibrous system. In conclusion, the results suggest that centrifugally spun flaxseed gum/PEO fibers enriched with kale extract have strong potential as sustainable antioxidant-active packaging materials for lipid-containing foods. Although the developed fibers demonstrated promising antioxidant and oxidation inhibitory performance in olive oil systems, future studies should investigate their effectiveness in more complex food matrices and under commercial storage conditions.

## Figures and Tables

**Table 1:** Viscosity, average diameter, water vapor permeability (WVP) values of centrifugally spun fibers.

Samples	Viscosity (Pa.s)	Average Diameter (µm)	WVP × 10^−12^ (g·m^−1^·s^−1^·Pa^−1^)
PGKE-0	0.132 ± 0.017 ^b^	1778.4 ± 21.5 ^c^	4.78 ± 0.191 ^b^
PGKE-1	0.209 ± 0.001 ^a^	2029.2 ± 2.26 ^b^	6.43 ± 0.085 ^a^
PGKE-3	0.19 ± 0.005 ^a^	2066.7 ± 14.8 ^b^	6.17 ± 0.057 ^a^
PGKE-5	0.178 ± 0.001 ^a^	2143.1 ± 6.08 ^a^	6.66 ± 0.375 ^a^

Columns with different letters are significantly different (*p*  ≤  0.05).

**Table 2:** Loading capacity (LC), antioxidant activity (AOA), encapsulation efficiency (EE), and total phenolic content (TPC) values of centrifugally spun fibers.

Samples	TPC (mg GAE/g)	AOA (%)	EE (%)	LC (%)
PGKE-0	0.067 ± 0.003 ^d^	0.748 ± 0.006 ^d^	N.D.	N.D.
PGKE-1	0.322 ± 0.004 ^c^	10.444 ± 0.245 ^c^	95.78 ± 0.552 ^a^	0.72 ± 0.007 ^c^
PGKE-3	0.440 ± 0.016 ^b^	17.007 ± 0.139 ^b^	46.55 ± 0.516 ^b^	1.67 ± 0.035 ^b^
PGKE-5	0.553 ± 0.006 ^a^	29.097 ± 0.117 ^a^	32.32 ± 0.834 ^c^	2.61 ± 0.05 ^a^

Significant differences (*p* < 0.05) exist between columns with different letters. “Not Detected” is what N.D. signifies.

**Table 3:** Thermal degradation behavior and DSC properties of flaxseed gum/PEO fibers.

Samples	Stage I (50–100 °C) Mass Loss (%)	Stage II (330–480 °C) Mass Loss (%)	Total Mass Loss (%)	Residual Mass (%)	T_m_ (°C)	ΔH_m_ (J/g)
PGKE-0	N.D.	95.22	97.79	2.21	66.83	122.58
PGKE-1	1.51	90.38	93.24	6.76	56.40	20.914
PGKE-3	1.56	89.62	92.94	7.06	54.81	17.83
PGKE-5	1.48	89.89	92.70	7.30	52.84	18.52

“Not Detected” is what N.D. signifies.

## Data Availability

The original contributions presented in the study are included in the article, further inquiries can be directed to the corresponding author.
